# Surfactant Protein D Deficiency Aggravates Cigarette Smoke-Induced Lung Inflammation by Upregulation of Ceramide Synthesis

**DOI:** 10.3389/fimmu.2018.03013

**Published:** 2018-12-18

**Authors:** Bartosz Pilecki, Helle Wulf-Johansson, Christian Støttrup, Patricia Troest Jørgensen, Pascal Djiadeu, Anders Bathum Nexøe, Anders Schlosser, Søren Werner Karlskov Hansen, Jens Madsen, Howard William Clark, Claus Henrik Nielsen, Jørgen Vestbo, Nades Palaniyar, Uffe Holmskov, Grith Lykke Sorensen

**Affiliations:** ^1^Department of Cancer and Inflammation Research, Institute for Molecular Medicine, University of Southern Denmark, Odense, Denmark; ^2^Translational Medicine, Lung Innate Immunity Research Laboratory, The Hospital for Sick Children Research Institute, Toronto, ON, Canada; ^3^Department of Child Health, Sir Henry Wellcome Laboratories, Academic Unit for Clinical and Experimental Sciences, Faculty of Medicine, University of Southampton, Southampton, United Kingdom; ^4^Institute for Life Sciences, University of Southampton, Southampton, United Kingdom; ^5^National Institute for Health Research, Southampton Respiratory Biomedical Research Unit, Centre for Biomedical Research, University Hospital Southampton NHS Foundation Trust, Southampton, United Kingdom; ^6^Center for Rheumatology and Spine Diseases, Institute for Inflammation Research, Copenhagen University Hospital Rigshospitalet, Copenhagen, Denmark; ^7^Department of Respiratory Medicine, Odense University Hospital, Odense, Denmark; ^8^Division of Infection, Immunity and Respiratory Medicine, Manchester Academic Health Science Centre, Manchester University NHS Foundation Trust, Manchester, United Kingdom; ^9^Department of Laboratory Medicine and Pathobiology, and Institute of Medical Sciences, Faculty of Medicine, University of Toronto, Toronto, ON, Canada

**Keywords:** surfactant protein D (SP-D), cigarette smoke (CS), mouse models, ceramide, ceramide synthase

## Abstract

Cigarette smoke (CS) is the main cause of chronic obstructive pulmonary disease. Surfactant protein D (SP-D) is an important anti-inflammatory protein that regulates host immune defense in the lungs. Here, we investigated the role of SP-D in a murine model of CS-induced inflammation. Pulmonary SP-D localization and abundance was compared between smoker and non-smoker individuals. For *in vivo* studies, wildtype, and SP-D-deficient mice were exposed to CS for either 12 weeks or 3 days. Moreover, the effect of therapeutic administration of recombinant fragment of human SP-D on the acute CS-induced changes was evaluated. Pulmonary SP-D appeared with heterogenous expression in human smokers, while mouse lung SP-D was uniformly upregulated after CS exposure. We found that SP-D-deficient mice were more susceptible to CS-induced macrophage-rich airway inflammation. SP-D deficiency influenced local pro-inflammatory cytokine levels, with increased CCL3 and interleukin-6 but decreased CXCL1. Furthermore, CS exposure caused significant upregulation of pro-inflammatory ceramides and related ceramide synthase gene transcripts in SP-D-deficient mice compared to wildtype littermates. Administration of recombinant fragment of human SP-D (rfhSP-D) alleviated CS-induced macrophage infiltration and prevented induction of ceramide synthase gene expression. Finally, rfhSP-D treatment attenuated CS-induced human epithelial cell apoptosis *in vitro*. Our results indicate that SP-D deficiency aggravates CS-induced lung inflammation partly through regulation of ceramide synthesis and that local SP-D enrichment rescues CS-induced inflammation.

## Introduction

Chronic obstructive pulmonary disease (COPD) is a serious lung disease with increasing morbidity and mortality rates worldwide (1). COPD is characterized by irreversible airflow limitation caused by persistent inflammation and structural changes (2). The major risk factor for COPD development and progression is tobacco smoking (3). Experimental models have shown that cigarette smoke (CS) exposure induces pulmonary oxidative stress and local inflammation, mimicking many characteristics of COPD (4). As existing therapeutic approaches prove widely ineffective against CS-induced inflammation (5), there is an urgent need for the development of new anti-inflammatory treatments of COPD.

Surfactant protein D (SP-D) is a pulmonary collectin produced by alveolar type II cells and club cells within the respiratory tract. SP-D is an important innate immune molecule that exerts its anti-inflammatory actions through multiple mechanisms, such as direct antimicrobial activity, pathogen opsonization, induction of phagocytic properties in alveolar macrophages and regulation of soluble mediator production from both structural and immune cells [reviewed in 6]. In mice, targeted SP-D ablation is reported to cause spontaneous emphysema-like pathology (7). Lungs of SP-D-deficient mice show disturbances in surfactant homeostasis and accumulation of enlarged, foamy macrophages overproducing reactive oxygen species and pro-inflammatory factors (7). Interestingly, SP-D is upregulated by CS in murine airways, while it is decreased in human bronchoalveolar lavage fluid (BAL) and increased in serum of smokers and COPD patients (8–11). Moreover, single nucleotide polymorphisms in the *Stfpd* gene encoding SP-D have been associated with COPD in several independent cohorts (12), suggesting the involvement of SP-D in regulation of CS-induced pathology.

The exact role of SP-D in CS-induced inflammation remains unknown. We hypothesized that SP-D regulates CS-induced lung inflammation and that genetic ablation of SP-D leads to aggravation of CS-induced pathology. To address this issue, we subjected SP-D-deficient mice to subchronic and acute CS exposure. Furthermore, recombinant fragment of human SP-D (rfhSP-D) was administered to acutely exposed mice to investigate whether local delivery of exogenous SP-D has a potential to dampen CS-related inflammatory processes.

## Materials and Methods

### SP-D Staining of Human Lung Tissue

Sections of non-malignant lung tissues collected from patients undergoing pulmonary malignant tumor resection were immunostained against SP-D (antibody produced in-house, dilution 1:5,000) for 1 h and visualized with horseradish peroxidase-conjugated goat anti-rabbit secondary antibody and 3,3′-diaminobenzidine chromogen (both from Dako, Glostrup, Denmark). Subject characteristics are shown in Supplementary Table 1. The study was carried out in accordance with the recommendations of the Regional Scientific Ethics Committee for Southern Denmark with written informed consent from all subjects. All subjects gave written informed consent in accordance with the Declaration of Helsinki. The protocol was approved by the Regional Scientific Ethics Committee for Southern Denmark.

### Animals

Male C57BL/6N wild type (WT) and SP-D-deficient littermate mice (aged 8–10 weeks) (13) were used for all animal experiments. Mice were housed in macrolon type II cages containing aspen woodchip bedding (Tapvei, Brogaarden, Gentofte, Denmark) and nesting material (EnviroDri, Brogaarden, Gentofte, Denmark). The environment was controlled with respect to temperature (21–24°C) and illumination (12 h light/dark cycle with a 30 min sunset and dawn function). The animals had access to pelleted chow and water *ad libitum*. The Danish National Animal Ethics Committee approved all animal experiments (ref. no. 2012-15-2934-00525 and 2016-15-0201-01077).

### Genotyping

DNA was extracted from tail biopsies of 3-week-old mice using the REDExtract-N-AmpTM Tissue PCR kit (Sigma-Aldrich, St. Louis, MO, USA) according to the manufacturer's instructions. SP-D genotypes were identified by multiplex PCR using the 5′-GGTTTCTGAGATGGGAGTCGTG-3′ as the forward primer, and 5′-TGGGGCAGTGGATGGAGTGTGC-3′ and 5′-GTGGATGTGGAATGTGCGAG-3′ as the reverse primers recognizing the WT allele and the knockout allele, respectively.

### Recombinant Fragment of Human SP-D (rfhSP-D)

RfhSP-D is a recombinant homotrimeric fragment of human SP-D consisting of the carbohydrate recognition domain, alpha-helical coiled-coil neck domain plus a short segment of 8 Gly-Xaa-Yaa repeats from the collagen domain. The fragment was expressed and purified as previously described (14). Endotoxin level in rfhSP-D was 1 EU/ml as measured by the Limulus Amebocyte Lysate Assay (BioWhittaker, Lonza, Basel, Switzerland).

### CS Exposure Procedure and rfhSP-D Administration

A whole body CS exposure system was used and consisted of a time-set pinch valve (C Lee Machining, Horsham, UK), an exposure chamber (Teague Enterprises, CA, USA) and a total suspended particulate (TSP) sampling unit (Teague Enterprises). Mice were exposed to mainstream CS five consecutive days a week for 12 weeks (subchronic protocol) or for 3 consecutive days (acute protocol) using 3R4F cigarettes without filter (Tobacco Health Research Institute, University of Kentucky, Lexington, KY, USA). CS was generated using a negative pressure system (flow-rate set at 1,500 ml/min). Room air was continuously pumped into the chamber for the remaining period between puffs. Mice were subjected to two consecutive rounds of smoking lasting 50 min each, with a smoke-free interval of 30 min between exposures. TSP levels were monitored by sampling a known air volume from the chamber through a filter (Pallflex Air Monitoring Filters TX40HI20-WW, Life Sciences, Pall, Port Washington, NY, USA) to validate CS consistency within the chamber. The average TSP concentration in the chamber was 457 mg/m^3^. Blood samples from facial vein were collected every 4th week, and serum cotinine levels were assessed by ELISA (Calbiotech, Spring Valley, CA, USA) according to the manufacturer's instructions. Control, room air-exposed mice were housed together with CS-exposed mice except for the smoking periods and underwent the same anesthetic procedures.

In some acutely treated mice, rfhSP-D (15 μg in 50 μl PBS) was administered intranasally under light isoflurane anesthesia 1 h prior to each smoking period (Supplementary Figure 1). Control mice received intranasal PBS only.

### Lung Function Measurements

Twenty-four hour after the last exposure mice were anesthetized by intraperitoneal injection of ketamine (100 mg/kg) and xylazine (10 mg/kg), tracheostomized and connected to a computer-controlled small animal ventilator (Flexivent, SCIREQ, Montreal, Canada). Lung function parameters were measured with mechanical ventilation set at 150 breaths/min with a tidal volume of 10 ml/kg and a positive end-expiratory pressure of 3 cm H_2_O. For each parameter, a coefficient of determination of 0.95 was the lower limit for accepting a measurement.

Immediately after lung function testing the mice were sacrificed by cardiac puncture. Serum was isolated by centrifugation (3,000 g, 5 min) from coagulated blood.

### BAL Preparation

The lungs were lavaged four times with 0.5 ml ice-cold phosphate-buffered saline (PBS) without Ca^2+^ and Mg^2+^ (Gibco, Waltham, MA, USA). PBS was instilled into the lungs and allowed to equilibrate for at least 30 s before recovery. The four aliquots were pooled and centrifuged at 900 g for 10 min at 4°C, and the supernatants were stored at −80°C until further analysis. Cells were washed in Red Blood Cell Lysing Buffer Hybri-Max (Sigma) and resuspended in PBS. Total cell number was determined with a Z2 Beckman-Coulter particle counter. Afterwards cells were cytospun at 500 rpm for 5 min and stained with Hemacolor (Merck Millipore, Billerica, MA, USA). Differential cell count was performed in a blinded manner by two independent observers, with 200 counted cells/sample. Mean macrophage diameter was calculated in a blinded manner by measuring cell diameter from at least three random fields of vision/section using ImageJ software (15).

### Measurements of Endogenous SP-D and rfhSP-D in BAL

Endogenous SP-D and rfhSP-D levels in BAL were quantified by ELISA as previously described (16).

### Flow Cytometry Analysis of BAL Cell Apoptosis

Isolated BAL cells were stained with Dead Cell Apoptosis Kit (Thermo Fisher Scientific), containing annexin V-FITC and propidium iodide (PI), according to the manufacturer's instructions. Apoptotic cells were defined as annexin V-positive, PI-negative.

### Cytokine Measurements

A Bio-Plex mouse cytokine and chemokine panel (Bio-Rad, Copenhagen, Denmark) was used to quantify CCL2, CCL3, IL-1β, IFN-γ, and TNF in BAL after 12 weeks of CS exposure. The assay was performed according to the manufacturer's instructions. The analysis was performed with a Luminex 100 IS 2.3 system using the Bio-Plex Manager 4.1.1 software.

The levels of CCL2, CCL3, IL-1β, IL-6, IFN-γ, TNF, and KC in BAL were measured using commercial ELISA kits (R&D Systems, Minneapolis, MN, USA, and eBioscience, San Diego, CA, USA) according to the manufacturers' instructions.

### RNA Extraction and cDNA Synthesis

The lungs were snap-frozen in liquid nitrogen and stored in −80°C until further processing. Lung RNA was isolated using TRIzol reagent according to the manufacturer's instructions (Invitrogen). cDNA was synthesized using the QuantiTect Reverse Transcription Kit (Qiagen, Denmark) according to the manufacturer's introductions.

### Real-Time PCR

Real-time PCR (qPCR) was performed using Taqman Universal PCR Master Mix II and Taqman Gene Expression Assays (Applied Biosystems, Carlsbad, CA, USA) for the following genes: Sftpd (Mm00486060), Tbp (Mm00446971), Gapdh (Mm99999915), Cers2 (Mm01258345), CerS5 (Mm00510998), CerS6 (Mm00556165). Reactions were performed in duplicates on a StepOnePlus Real-Time PCR system. Relative mRNA levels were calculated using the 2^ΔΔ*CT*^ method. Tbp and Gapdh were used as endogenous controls.

### Lipid Analysis

Lipid analysis was performed at the Analytical Facility for Bioactive Molecules (The Hospital for Sick Children, Toronto, Canada). Samples were spiked with a mixture of internal standards (IS) (C17 base ceramide (10 ng) and PC, LPC (1 μg)). BAL samples (200 μl) and standards were extracted twice with 2:1 chloroform:methanol mixture. The lower chloroform layers were removed, combined, and then taken to dryness under a gentle flow of nitrogen gas. Residues were reconstituted in 100 μl 0.2% formic acid in ethanol and analyzed by LC-MS/MS. LC/MS/MS HPLC was performed using an Agilent 1200 Series HPLC (Agilent, Santa Clara, CA, USA). The analytical column was Kinetex C18 (100 × 2.1 mm, 2.6 μm particle size) from Phenomenex (Phenomenex, Torrance, CA). The HPLC mobile phases consisted of (A) = 0.05% formic acid in water/acetonitrile/methanol (2/1/1, v/v/v) and (B) = 0.05% formic acid in tetrahydrofuran/acetonitrile/methanol (2/1/1, v/v/v). A flow rate of 0.4 ml/minute was used with a gradient of solvent composition 40% B to 85% B, followed by re-equilibration to initial condition for 2 min. Each run lasted 19 min. The HPLC system was coupled to an AB SCIEX API4000 triple-quadrupole mass spectrometry (SCIEX, Framingham, MA, USA). Positive electro spray ionization (ESI) multiple reaction monitoring (MRM) was used. Data were collected and analyzed using Analyst v.1.5.1 (SCIEX).

### Western Blotting

Frozen lung tissues were homogenized in RIPA buffer (Sigma) containing protease inhibitors (Complete tablets, Roche, Indianapolis, IN, USA). Samples were equalized according to the protein concentration, boiled for 1 min in 4x loading buffer, separated by SDS-PAGE on NuPage 4–12% Bis-Tris gels (Bio-rad, Hercules, CA, USA) and transferred onto polyvinylidene fluoride membranes using the semidry transfer system (Bio-rad). The membranes were then blocked for 2 h at room temperature with 5% nonfat dry milk (Bio-rad) in Tris-buffered containing 0.5 M NaCl and 0.1% Tween (TBST), incubated with primary antibodies against GAPDH (1:500, Santa Cruz) or LC3B (1:5000, Sigma) overnight at 4°C, washed with TBST and incubated with goat anti-rabbit Ig-HRP or rabbit anti-mouse Ig-HRP secondary antibody (1:5000 and 1:10000, respectively, both from Dako, Glostrup, Denmark). Protein expression was detected using ECL reagents and quantified by densitometry using Image Studio Lite software (LI-COR Biotechnology).

### Cigarette Smoke Extract (CSE) Preparation

CSE was prepared by bubbling smoke from one 3R4F research cigarette without filter into a Buchner flask containing 10 ml of RPMI-1640 medium (Thermo Fisher Scientific, Waltham, MA, USA). The smoke was drawn into the medium under vacuum over a period of 1–2 min. CSE was then filtered through 0.22 μm filter (GE Healthcare, Broendby, Denmark) and used immediately. Medium bubbled with room air was used as a control.

### Apoptosis Assay

Human adenocarcinoma alveolar basal epithelial A549 epithelial cells (ATCC) were cultured in RPMI-1640 medium supplemented with 10% fetal bovine serum, 2 mM L-glutamine, 50 U/ml penicillin and 50 μg/ml streptomycin (all from Gibco). Cells were seeded in 96-well plates at the density of 10,000 cells/well. After 8 h of incubation allowing for the attachment, cells were serum-starved overnight and stimulated with 2.5% CSE for 24 h. In some experiments, cells were pre-treated with 20 μg/ml rfhSP-D 1 h before and throughout CSE stimulation. Cell apoptosis was assessed by Caspase-Glo®3/7 Assay (Promega) according to the manufacturer's instructions.

### Statistical Analysis

Statistical analysis was performed using Prism 7 software (GraphPad, La Jolla, California, USA). Data are presented as means + SEM. Parametric results were analyzed using Student's *t*-test or one-way analysis of variance (ANOVA) followed by Bonferroni's multiple comparisons test. Correlations were analyzed with Pearson's test. A *p* < 0.05 was considered statistically significant.

## Results

### Endogenous SP-D Is Upregulated by CS

**Figure 1:**
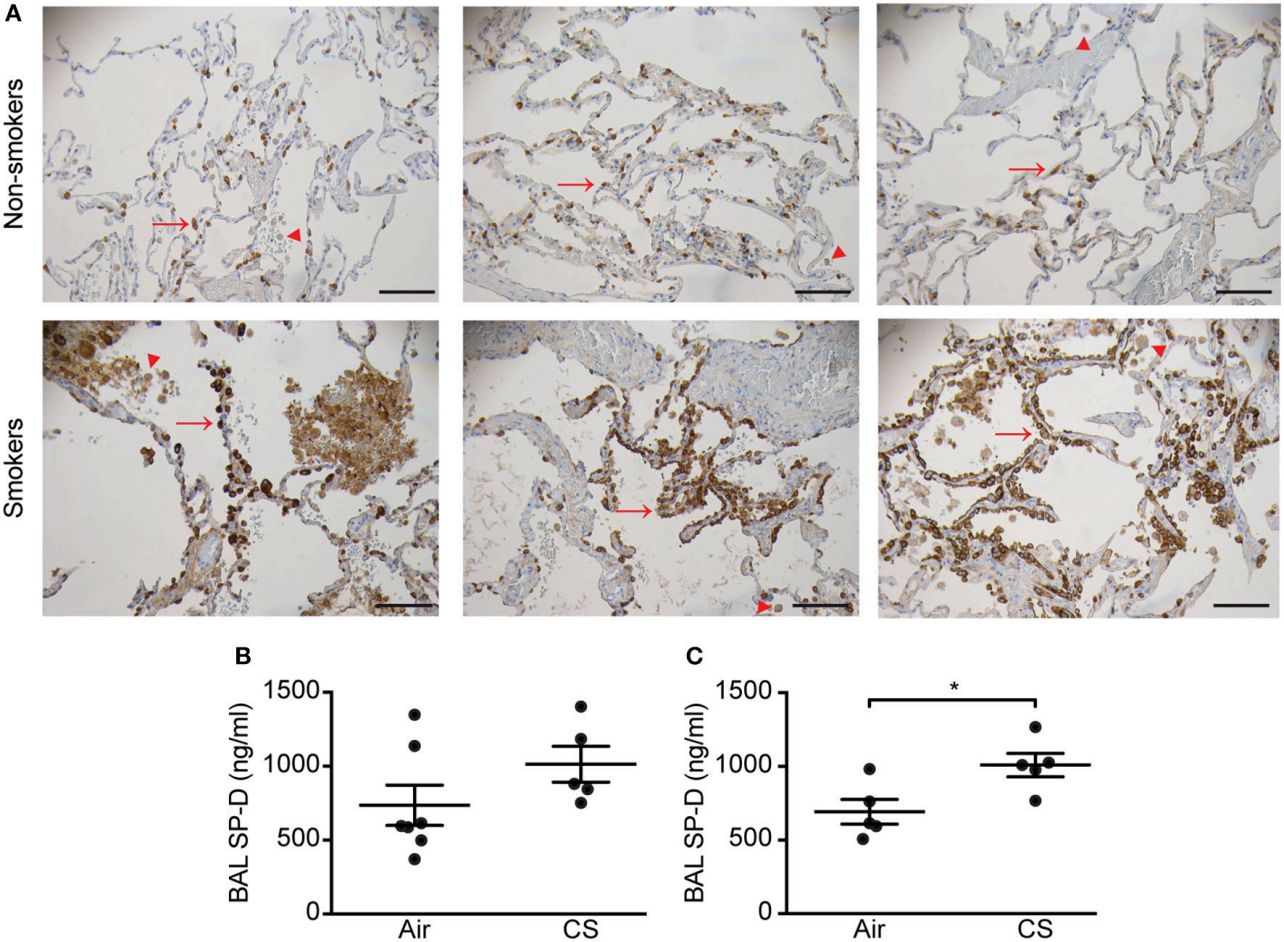
Regulation of pulmonary SP-D levels by cigarette smoke (CS) in humans and mice. **(A)** Human SP-D is upregulated in the lungs of smokers compared to non-smoker controls. Each picture represents a single subject. Examples of alveolar type II cells and alveolar macrophages are marked with red arrows and arrowheads, respectively. Representative pictures are shown. Scale bar, 100 μm. **(B,C)** Endogenous murine SP-D is upregulated in bronchoalveolar lavage fluid (BAL) after **(B)** 12 weeks and **(C)** 3 days of CS exposure. *n* = 5–7. ^*^p < 0.05, analyzed by Student's *t*-test.

Initially, we analyzed the SP-D staining pattern in human lung tissue. In the lungs of non-smokers, SP-D was localized to alveolar type II cells and weakly to alveolar macrophages. By contrast, lungs from smokers exhibited focal areas rich in alveolar type II cells strongly positive for SP-D (Figure 1A), while areas with marked tissue loss appeared with low SP-D expression similar to that of non-smoker lungs (not shown). Alveolar macrophages from smokers appeared with more intense immunostaining for SP-D than from non-smoking individuals (Figure 1A).

We then analyzed possible CS-dependent regulation of SP-D expression in mice. Similar to previous findings (8), endogenous SP-D levels were elevated in BAL of WT mice exposed to CS for 12 weeks or 3 days (Figures 1B,C). We did not detect SP-D upregulation by CS on the mRNA level (Supplementary Figure 2). Mean serum cotinine levels were comparable between CS-exposed WT and SP-D-deficient mice (207.7 ± 32.3 ng/ml and 154.9 ± 43 ng/ml, respectively) and remained undetectable in control animals.

### SP-D Deficiency Causes Alterations in Lung Mechanics Similar to CS-Induced Changes

**Figure 2:**
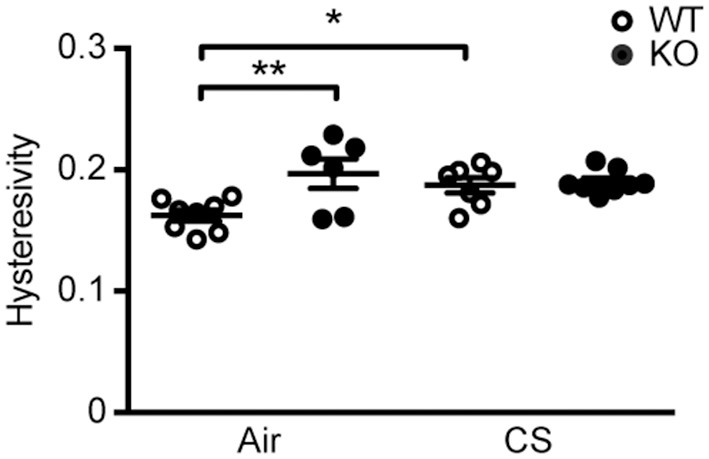
SP-D deficiency and cigarette smoke (CS) exposure cause changes in pulmonary mechanics in mice. Changes in lung hysteresivity after 12 weeks of CS exposure. *n* = 6–8. ^*^p < 0.05, ^**^p < 0.01, analyzed by one-way ANOVA followed by Bonferroni's test.

We performed invasive lung function testing to assess potential aggravating effects of prolonged CS exposure on mechanical properties of SP-D-deficient lungs. Subchronic CS exposure had only minor effects on lung function parameters (Supplementary Figure 3), possibly because the experimental protocol was too short when using the C57BL/6 strain that is only mildly susceptible to CS-induced emphysema (4). Both inspiratory capacity and the estimate for total lung capacity were significantly increased by CS exposure in both genotypes (Supplementary Figures 3G,H). Interestingly, we also found that lung hysteresivity was increased in naïve SP-D-deficient mice to the level seen in CS-exposed WT mice, and was not increased further after CS (Figure 2). Hysteresivity is defined as a ratio between tissue damping and tissue elastance (Supplementary Figures 3D,E), parameters describing energy dissipation and energy conservation in the alveoli, respectively. Increased lung hysteresivity reflects pulmonary structural and functional abnormalities (17), which indicates that air space enlargement induced by SP-D deficiency causes disruption of the viscoelastic mechanical balance of the lungs.

### SP-D Deficiency Aggravates Airway Macrophage Inflammation Caused by CS

**Figure 3:**
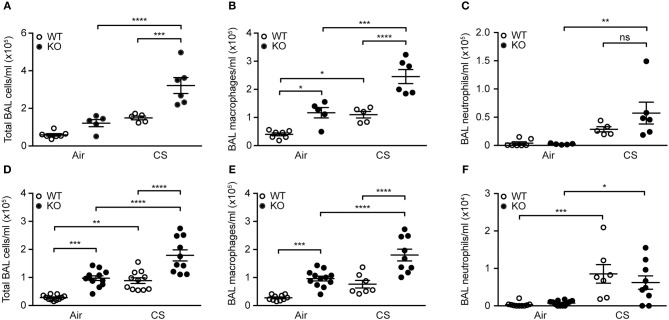
SP-D deficiency aggravates cigarette smoke (CS)-induced airway inflammation in mice. SP-D-deficient mice exhibit higher total cell count and macrophage number in bronchoalveolar lavage fluid (BAL) compared to WT littermates both after **(A,B)** 12 weeks and **(D,E)** 3 days of CS exposure, while the increase in neutrophil numbers induced by **(C)** 12 weeks, or **(F)** 3 days of CS exposure is not influenced by SP-D deficiency. *n* = 5–7 **(A–C)**, 7–12 **(D–F)**. ^*^p < 0.05, ^**^ *p* < 0.01,^***^ *p* < 0.001, ^****^ *p* < 0.0001, analyzed by one-way ANOVA followed by Bonferroni's test. ns, not significant.

Mice exposed to CS for 12 weeks displayed higher total number of BAL cells, specifically macrophages and neutrophils. The increase in macrophage number was significantly higher in SP-D-deficient mice than in WT littermates (Figures 3A–C). We observed similar inflammatory pattern in the airways of acutely smoked mice, where CS exposure for 3 days caused the increase in BAL neutrophil and macrophage numbers, the latter being significantly higher in SP-D-deficient mice compared to WT littermates (Figures 3D–F). As expected, naïve SP-D-deficient mice exposed to room air had higher number of BAL macrophages than age-matched WT mice.

### Treatment With RfhSP-D Ameliorates Acute CS-Induced Airway Macrophage Inflammation

**Figure 4:**
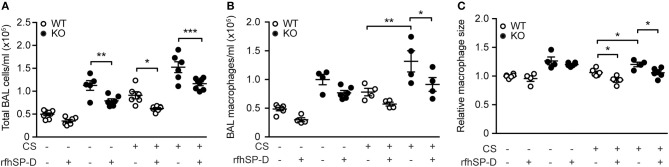
Therapeutic administration of recombinant SP-D fragment attenuates airway inflammation caused by acute cigarette smoke (CS) exposure in mice. Treatment with recombinant fragment of human SP-D (rfhSP-D) lowers **(A)** total cell number, **(B)** macrophage number, and **(C)** macrophage size in bronchoalveolar lavage fluid (BAL) of CS-exposed mice. *n* = 4–8. ^*^p < 0.05, ^**^p < 0.01, ^***^p < 0.001, analyzed by one-way ANOVA followed by Bonferroni's test.

We then investigated if administration of rfhSP-D could rescue the inflammatory phenotype of SP-D-deficient mice seen after 3 days of CS exposure. Both total cell count and macrophage numbers in BAL were significantly attenuated in mice treated with rfhSP-D, while the number of neutrophils remained unaffected (Figures 4A,B and data not shown). Moreover, rfhSP-D administration to CS-exposed mice resulted in normalization of BAL macrophage size (Figure 4C). Importantly, rfhSP-D reduced local macrophage inflammation in all experimental groups (Figures 4A,B). Administration of rfhSP-D had no effect on endogenous SP-D secretion (data not shown).

### Regulation of Pulmonary Pro-Inflammatory Mediators After CS Exposure

**Figure 5:**
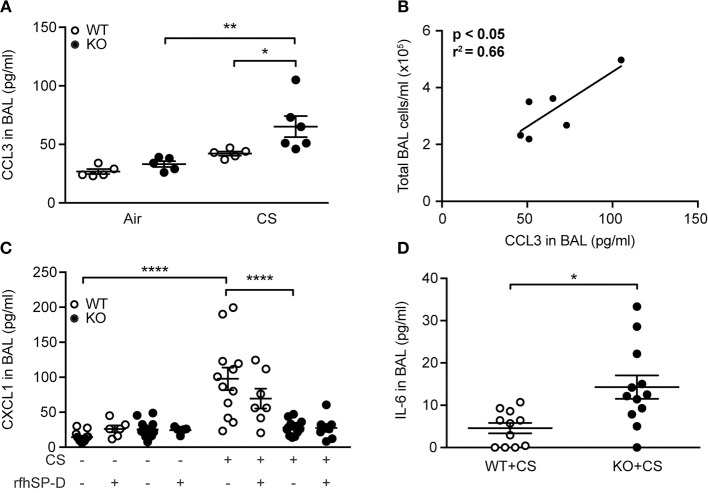
SP-D-dependent regulation of cigarette smoke (CS)-induced pro-inflammatory cytokines in mice. **(A)** CCL3 levels in bronchoalveolar lavage fluid (BAL) are significantly increased in SP-D-deficient mice compared to WT littermates after subchronic CS exposure. **(B)** Significant correlation between BAL levels of CCL3 and total BAL cell count in CS-exposed SP-D-deficient mice. **(C)** SP-D-deficient mice fail to upregulate CXCL1 in BAL after acute CS exposure. (D) BAL levels of IL-6 after acute CS exposure. *n* = 5–13. ^*^p < 0.05, ^**^p < 0.01, ^****^p < 0.0001, analyzed by **(A,C)** one-way ANOVA followed by Bonferroni's test or **(D)** Student's *t*-test.

To determine if modulation of cytokine levels could explain the differences observed in inflammatory cell content, we investigated the airway cytokine profile of CS-exposed mice. We did not observe changes in BAL levels of interleukin (IL)-1β, interferon (IFN)-γ and tumor necrosis factor (TNF) between groups exposed to CS for 12 weeks (data not shown), while CCL2 was increased independently of SP-D genotype (Supplementary Table 2). However, CS-treated SP-D-deficient mice showed significantly elevated BAL levels of macrophage chemoattractant CCL3 compared to WT littermates (Figure 5A). Moreover, CCL3 levels in CS-exposed SP-D-deficient mice were significantly correlated with BAL cell count, suggesting its role in macrophage attraction to the SP-D-deficient lung (Figure 5B).

Acute CS exposure did not influence IL-1β, IFN-γ, or TNF levels (data not shown), while CCL2 and CCL3 were only modestly increased independent of SP-D genotype (Supplementary Table 2). However, we detected significant differences in regulation of CXCL1, which was upregulated after CS in WT but not in SP-D-deficient mice (Figure 5C). Furthermore, BAL levels of IL-6 were significantly higher in SP-D-deficient mice than WT littermates following acute CS exposure (Figure 5D).

### CS Induces Upregulation of Ceramides as Well as Ceramide Synthase (CerS) Genes in SP-D-Deficient Mice That Is Reversed by RfhSP-D

Next, we used mass spectrometry (MS) to analyze the lipid profile in BAL of CS-exposed mice. As expected, SP-D-deficient mice showed elevated BAL phospholipid pool (Supplementary Table 3). The levels of analyzed phospholipids were not influenced by CS exposure (Supplementary Table 3).

**Figure 6:**
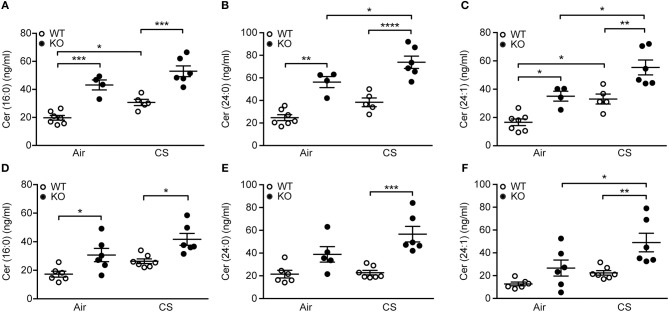
Upregulation of pulmonary ceramides in cigarette smoke (CS)-exposed SP-D-deficient mice. SP-D-deficient mice show higher levels of long-chain and very long-chain ceramides in bronchoalveolar lavage fluid (BAL) after **(A–C)** 12 weeks and **(D–F)** 3 days of CS exposure compared to WT littermates. *n* = 4–7. ^*^p < 0.05, ^**^p < 0.01, ^***^p < 0.001, ^****^p < 0.0001, analyzed by one-way ANOVA followed by Bonferroni's test.

Furthermore, we observed significant differences in pro-inflammatory long-chain and very long-chain ceramides, which were upregulated by CS but, importantly, also by SP-D deficiency (Figures 6A–C). These changes were detectable already after 3 days of CS exposure (Figures 6D–F). Levels of corresponding dihydroceramide species were also the highest in CS-exposed SP-D-deficient mice (Supplementary Figure 4).

**Figure 7:**
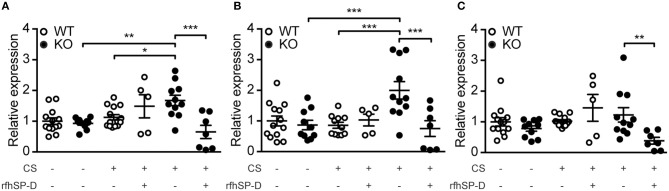
Recombinant SP-D administration reverses pulmonary overexpression of ceramide synthases (CerS) in cigarette smoke (CS)-exposed mice. Lung mRNA levels of **(A)** CerS2, **(B)** CerS5, and **(C)** CerS6 are increased after acute CS exposure in SP-D-deficient mice and are normalized by treatment with recombinant fragment of human SP-D (rfhSP-D). *n* = 5–14. ^*^p < 0.05, ^**^p < 0.01, ^***^p < 0.001, analyzed by one-way ANOVA followed by Bonferroni's test.

To follow up on these observations, we investigated the expression pattern of genes responsible for ceramide synthesis. We observed that CS exposure induced upregulation of CerS2 and CerS5 in SP-D-deficient mice but not in WT littermates (Figures 7A,B). Moreover, administration of rfhSP-D was able to normalize the expression of CerS2 and CerS5 to control levels (Figures 7A,B) as well as downregulate the related enzyme CerS6 (Figure 7C).

### Effect of SP-D Deficiency and RfhSP-D on CS-Induced Cell Death

**Figure 8:**
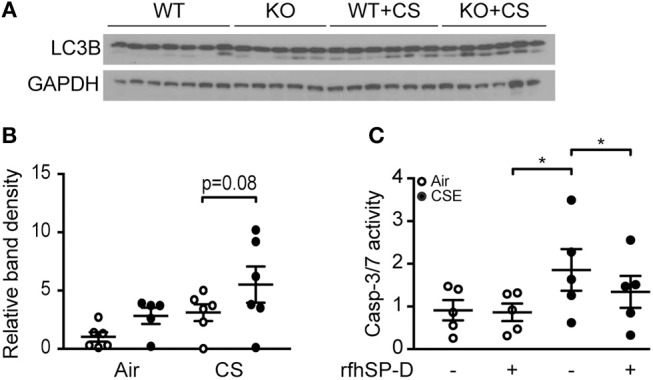
The effect of endogenous and exogenous SP-D on cigarette smoke (CS)-induced human epithelial cell death. **(A,B)** Autophagy marker LC3B-II (lower band) is increased in lung homogenates of SP-D-deficient mice after 12 weeks of CS exposure. Each lane in **(A)** represents a single lung homogenate sample, and the relative band density is quantified in **(B)**. **(C)** *In vitro* administration of recombinant fragment of human SP-D (rfhSP-D) attenuates cigarette smoke extract (CSE)-induced apoptosis of A549 alveolar epithelial cells. The cells were pre-treated with 20 μg/ml rfhSP-D for 1 h before stimulation with 2.5% CSE for 24 h. Apoptosis was measured as relative caspase-3/7 activity. Data are means of 5 independent experiments. ^*^p < 0.05, analyzed by one-way ANOVA followed by Bonferroni's test.

As CS and subsequent ceramide accumulation may lead to alveolar cell death through various pathways, such as autophagy and apoptosis (18), we investigated if SP-D genotype influences these processes. SP-D-deficient mice subjected to subchronic CS exposure showed increased autophagy, detected as increase in abundance of lipidated, active form of the crucial autophagy protein LC3B (LC3B-II) within the lung, compared to CS-exposed WT littermates (Figures 8A,B).

Finally, we co-stimulated human alveolar epithelial A549 cells with CSE and rfhSP-D to investigate if exogenous SP-D can be protective against CSE-triggered toxicity. CSE induced caspase-dependent apoptosis in A549 cells at low concentrations and was highly cytotoxic at concentrations above 5% (data not shown). Administration of rfhSP-D attenuated apoptosis measured as caspase-3/7 activation caused by 2.5% CSE stimulation (Figure 8C).

## Discussion

In this study, we evaluated the role of SP-D in the development of CS-induced inflammation. We showed that SP-D appeared with focally increased expression in CS-exposed human lungs. We further demonstrated that SP-D-deficient mice are susceptible to macrophage-rich airway inflammation and pro-inflammatory ceramide accumulation caused by CS exposure, and that rfhSP-D treatment reverses macrophage accumulation as well as upregulation of CerS genes that are crucial for ceramide generation. Finally, we also showed that SP-D-deficient mice exhibit elevated pulmonary levels of autophagy markers after prolonged CS exposure and that rfhSP-D attenuates CSE-induced epithelial cell apoptosis *in vitro*. Collectively, our results describe SP-D as an important regulator of CS-induced pulmonary inflammation.

Both local and circulatory SP-D levels are disturbed in smokers and COPD patients, who exhibit vast induction of serum SP-D with a concomitant reduction of BAL SP-D (9, 19). This has been attributed to increased leakage of pulmonary SP-D from the alveolar space into blood (20). High serum SP-D has been linked to increased COPD mortality (21). It has also been shown to inversely correlate with lung function parameters (19) and its levels further rise during COPD exacerbations, possibly reflecting local compensatory upregulation of pulmonary SP-D (22) or further exaggerated alveolar-capillary leakage. Here we show that SP-D is focally upregulated in the alveolar epithelium and macrophages of smokers, most probably as a local compensatory mechanism aimed for cellular protection against CS-induced damage. This response seems heterogenous across the lung parenchyma, as areas of low tissue density showed SP-D expression levels similar to that observed in non-smoker lungs.

Contrary to the clinical situation described above, endogenous murine SP-D levels were elevated in BAL after both subchronic and acute CS exposure, which is in agreement with a previous study of chronic, 6-months-long CS challenge (8). As SP-D is also upregulated in BAL in other disease models, such as LPS- or bleomycin-induced inflammation, such compensatory increase in pulmonary SP-D seems as a common sign of lung injury in the mouse (23). Although we have not detected previously reported changes in SP-D mRNA expression after CS exposure (8), differences in mouse strain (previously used B6C3F1 mice are extremely sensitive to CS exposure), type of cigarettes and exposure time might explain why the CS-related effects on SP-D mRNA levels were more pronounced in the previous study.

Effects of CS exposure in mice are highly strain-dependent. BALB/c mice are susceptible to CS-induced pathology characterized by potent neutrophil recruitment and cytokine upregulation, while CS exposure of relatively resistant C57BL/6 strain results in macrophage-rich infiltration and a limited increase of pro-inflammatory mediators (4, 24). Accordingly, we detected only minor changes in BAL pro-inflammatory cytokine profile after CS exposure. Nevertheless, levels of CCL3 and IL-6 were significantly increased due to SP-D deficiency. CCL3 is a potent monocyte and macrophage chemoattractant (25) that is increased in sputum of COPD subjects (26). We showed that BAL CCL3 levels are elevated in SP-D-deficient mice and positively correlate with the airway inflammatory cell number, which underlines its importance in CS-induced mononuclear cell recruitment to the SP-D-deficient lung. IL-6 is a pro-inflammatory cytokine implicated in the pathogenesis of COPD (27, 28) and previously reported to be upregulated in SP-D-deficient mice (29). Interestingly, IL-6 seems to regulate CCL3 expression after pulmonary insult (30). We suggest that increased CCL3 is responsible for macrophage influx to the airways in SP-D-deficient mice, possibly through an IL-6-dependent mechanism that remains to be elucidated.

Unexpectedly, we observed that CS-exposed SP-D-deficient mice failed to upregulate CXCL1, a canonical neutrophil chemoattractant. In line with our findings, absence of CXCL1 upregulation has also been recently reported in SP-A/SP-D double-deficient mice subjected to urinary tract infection (31). Apart from its anti-inflammatory functions mediated by the recognition of the C-terminal domain, full-length SP-D is also capable of mounting pro-inflammatory responses through interaction between its N-terminal collagenous tail and calreticulin/CD91 receptor complex (32), which might explain why CXCL1 is not generated after CS in SP-D-deficient mice. However, presence of CXCL1 was not critical for pulmonary neutrophil recruitment in our study, as BAL neutrophil numbers were similar in CS-exposed WT and SP-D-deficient mice.

SP-D deficiency causes excessive accumulation of surfactant phospholipids (33). We performed detailed MS-based analysis and showed that CS exposure has no impact on BAL phospholipid pool. However, we observed that ceramides, particularly the most abundant long- and very long-chain species (16:0), (24:0) and (24:1), respectively, are upregulated in SP-D-deficient mice. Thus, we are, to our best knowledge, the first to report SP-D involvement in ceramide biology. Ceramides are bioactive sphingolipids with pro-inflammatory and pro-apoptotic properties that are increased by CS exposure *in vivo*, leading to alveolar and endothelial cell death and subsequently emphysema (34, 35). Ceramide can be generated by three distinct pathways: *de novo* formation, salvage from sphingosine or sphingomyelinase-dependent sphingomyelin cleavage [reviewed in (36)]. We also detected increases in corresponding dihydroceramide species that are direct ceramide precursors in the *de novo* pathway, which suggests *de novo* synthesis as a potential mechanism of ceramide upregulation in CS-exposed SP-D-deficient mice.

The family of CerS enzymes, responsible for the crucial step of *de novo* ceramide synthesis, contains six members with distinct substrate specificity. CerS2 uses C22-C24 fatty acyl chains, while CerS5/CerS6 use mostly C16 to generate very long-chain and long-chain ceramides, respectively; these are also the most abundant pulmonary CerS isoforms (37). The balance between ceramide species is crucial for maintaining lung homeostasis, underlined by the phenotype of CerS2-null mice that show accumulation of foamy alveolar macrophages and increase in total lung volume, possibly caused by strong compensatory accumulation of C16-ceramide (37). Both C16- and C24-ceramides have been implicated in pro-apoptotic signaling, leading to caspase-3 activation (38, 39). We showed that SP-D deficiency renders lungs susceptible to CS-induced upregulation of relevant CerS that can be reversed by rfhSP-D. SP-D-mediated inhibition of p38-induced stress response (32) is likely responsible for this effect. However, this was not explored further, and future studies are needed to clarify the exact mechanisms behind rfhSP-D regulation of ceramide synthesis.

Besides apoptosis, other types of cell death including autophagy have been recently linked to CS-induced lung injury and COPD pathogenesis (40), and long chain ceramides have been shown to mediate this response (41). We have found that SP-D deficiency aggravates the increase in active autophagy protein after prolonged CS exposure, further strengthening the hypothesis that SP-D participates in controlling CS-induced pathology through regulation of ceramides and their downstream effects on cellular stress.

As the 3-months-long subchronic CS exposure was not sufficient to induce a marked loss of alveolar tissue in SP-D-deficient mice, we did not pursue investigation of epithelial apoptosis *in vivo*. Instead, such effects were investigated in human epithelial cells *in vitro*. We found that rfhSP-D attenuated effector caspase activation in A549 cells exposed to low dose CSE. This implicates that SP-D protects epithelial cells from CSE-induced apoptosis. Ceramide accumulation has been previously reported to be responsible for caspase-dependent apoptosis in lung epithelial cells (42). Thus, SP-D-dependent downregulation of ceramide generation might be responsible for the observed epithelial protection from apoptosis in cells treated with rfhSP-D. Of note, protective effects of murine SP-D overexpression on CS condensate-induced toxicity have been previously reported in A549 cells (8) and SP-D has been shown to inhibit caspase-3 activation (43, 44). On the other hand, SP-D-mediated effects on apoptosis seem to be context-specific, with recent reports indicating that SP-D can actually induce apoptosis in cancer cell lines (45–47). The possible mechanistic link between SP-D-induced ceramide inhibition and alveolar epithelium protection remains to be investigated in further details.

Immunomodulatory functions of SP-D have been highlighted by several therapy-based animal studies that used local installation of exogenous SP-D. Full-length human SP-D administration protected against ventilation-induced lung injury and inflammation in premature newborn lambs (48), while rfhSP-D has been reported to attenuate allergic asthma (49). In agreement with this, we showed that rfhSP-D treatment attenuates CS-induced macrophage infiltration, emphasizing its protective potential. Importantly, the protective effects of rfhSP-D were also present in WT mice, which suggests that although we observed upregulation of endogenous SP-D in WT mice, this compensatory increase in SP-D production is itself not sufficient to control CS-induced inflammation. Compared to native SP-D, rfhSP-D contains only a small collagen chain, a coiled-coil region and a carbohydrate recognition domain. Despite lacking the collagenous tail, rfhSP-D seems fully capable of mounting anti-inflammatory responses, previously suggested to be a consequence of the binding between its C-terminal carbohydrate recognition domain and SIRP-α receptor on resident cells, leading to inhibition of NF-κB signaling (32). Importantly, increased endogenous SP-D levels have been recently shown to confer protection against COPD risk and lung function decline, which supports SP-D as a very promising therapeutic target (50) and warrants further studies on SP-D-based therapy.

A limitation of our study is that we used a single rfhSP-D concentration. However, more detailed dose response analysis would help to establish the optimal conditions of rfhSP-D treatment. Furthermore, the CS exposure model mimics the inflammatory component of COPD only to a limited extent and may only reflect early events in CS-induced pulmonary injury seen in humans, before a significant loss of alveolar tissue occurs. As our SP-D-deficient mice were on the C57BL/6 background that is relatively resistant to CS-induced emphysema, a much longer CS exposure than the used 3-months-long subchronic model would be necessary to provoke significant changes in pulmonary architecture.

In conclusion, we have shown that SP-D deficiency aggravates airway macrophage inflammation and ceramide accumulation caused by CS exposure, and that local rfhSP-D administration attenuates CS-induced inflammation and ceramide synthesis as well as epithelial cell apoptosis. Thus, we have identified a possible novel mechanism by which SP-D protects against pulmonary pathology triggered by CS exposure.

## Author Contributions

BP and HW-J performed experiments, analyzed data, and wrote the manuscript. CS, PJ, PD, AN, AS, SH, JM, HC, CN, and NP participated in data collection and analysis. JV and UH participated in study conception. CS, SH, JV, NP, UH, and GS reviewed the manuscript. All authors revised the final version of the manuscript. GS conceived and supervised the study. BP and GS are the guarantors responsible for the overall content of the manuscript.

### Conflict of Interest Statement

JM and HC have a patent pending, no PCT/GB2016/054004. JV reports personal fees from GlaxoSmithKline, personal fees from Chiesi Pharmaceuticals, personal fees from Boehringer-Ingelheim, personal fees from Novartis, personal fees from AstraZeneca, all outside the submitted work. The remaining authors declare that the research was conducted in the absence of any commercial or financial relationships that could be construed as a potential conflict of interest.
