# Supplementary material for: Surfactant Protein D Deficiency Aggravates Cigarette Smoke-Induced Lung Inflammation by Upregulation of Ceramide Synthesis
Source: Front Immunol. 2018 Dec 18;9:3013. doi: 10.3389/fimmu.2018.03013 (PMC6305334; doi:10.3389/fimmu.2018.03013)
Supplement: Supplementary file 1 [file Data_Sheet_1.docx]

**ONLINE DATA SUPPLEMENT**

| Patient ID | Sex | Age | Smoking Status | Pack years |
| --- | --- | --- | --- | --- |
| 16ouh052660 | Female | 58 | Non-smoker | - |
| 15ouh015278 | Female | 71 | Non-smoker | - |
| 15ouh025164 | Female | 67 | Non-smoker | - |
| 15ouh022522 | Female | 50 | Non-smoker | - |
| 15ouh049048 | Female | 50 | Smoker | 100 |
| 15ouh000276 | Female | 73 | Smoker | 30 |
| 15ouh058909 | Male | 50 | Smoker | 72 |

Supplementary Table 1. Subject characteristics. Non-smokers were never-smokers.

| ­ | Subchronic | | | | Acute | | | |
| --- | --- | --- | --- | --- | --- | --- | --- | --- |
|  | Air | | CS | | Air | | CS | |
| Soluble mediator | WT | KO | WT | KO | WT | KO | WT | KO |
| CCL2 | 19.6(2.4) | 21.8(3.6) | 58.8(9.1)* | 78(16.63)** | 9.9(7) | 14.1(4.8) | 20.9(11.2) | 26(7.8) |
| CCL3 |  |  |  |  | 13.1(1.7) | 14(2.5) | 25.1(13.9) | 23.8(5.2) |

Supplementary Table 2. CCL2 and CCL3 levels in WT and SP-D-deficient mice after cigarette smoke (CS) exposure. Cytokine levels were measured in bronchoalveolar lavage fluid after 12 weeks (subchronic) or 3 days (acute) of CS exposure. Data are shown as pg/ml and are presented as means(SEM), n = 3-6. *p<0.05, **p<0.01 for genotype-matched CS groups versus air control groups, analyzed by one-way ANOVA followed by Bonferroni’s test. Levels of CCL3 after subchronic CS exposure are presented in Fig. 5A.

|  | Air | | CS | | |
| --- | --- | --- | --- | --- | --- |
| Phospholipid species | WT | KO | WT | KO |  |
| Subchronic (μg/ml) |  |  |  |  |  |
| (16:0/16:0) | 82.1(5.6) | 240.5(11.4)**** | 87.1(5.9) | 211.3(14.7)**** |  |
| (16:0/14:0) | 9.9(0.8) | 32.7(2.1)**** | 12.4(1.1) | 32.8(2.9)**** |  |
|  |  |  |  |  |  |
| Acute (μg/ml) |  |  |  |  |  |
| (16:0/16:0) | 67.8(5.7) | 171.9(22.6)*** | 48.9(4.3) | 152.2(15.3)**** |  |
|  |  |  |  |  |  |
| (16:0/14:0) | 11.8(0.8) | 38.7(6.7)*** | 8.7(0.8) | 34.6(4.6)*** |  |
|  |  |  |  |  |  |
| (16:0/18:0) | 1.65(0.3) | 5.8(1.5)** | 1.0(0.1) | 4.0(0.6)* |  |
|  |  |  |  |  |  |
| (16:0/18:1) | 9.7(0.9) | 32.3(5.4)*** | 8.0(0.4) | 29.5(3.5)*** |  |
|  |  |  |  |  |  |
| (16:0/18:2) | 11.3(1.1) | 35.2(7.3)** | 6.6(0.7) | 28.5(3.0)** |  |
|  |  |  |  |  |  |
| (18:0/18:2) | 0.8(0.1) | 2.1(0.6)* | 0.6(0.1) | 1.6(0.2) |  |
|  |  |  |  |  |  |
| (16:0/20:4) | 2.9(0.2) | 9.2(1.5)*** | 2.2(0.2) | 8.8(1.1)*** |  |
|  |  |  |  |  |  |
| (18:0/20:4) | 0.7(0.1) | 1.9(0.4)** | 0.6(0.1) | 1.7(0.2)** |  |
| (16:0/22:6) | 3.4(0.3) | 9.7(1.6)*** | 2.1(0.2) | 7.8(0.8)*** |  |
|  |  |  |  |  |  |
| (18:0/22:6) | 0.4(0.1) | 0.9(0.2)* | 0.3(0.01) | 0.8(0.9)** |  |
|  |  |  |  |  |  |

Supplementary Table 3. Phospholipid content in WT and SP-D deficient mice after cigarette smoke (CS) exposure. Phospholipids were measured in bronchoalveolar lavage fluid by mass spectrometry. Data are presented as means(SEM), n = 4-7. *p<0.05, **p<0.01, ***p<0.001, ****p<0.0001 for KO versus treatment-matched WT groups, analyzed by one-way ANOVA followed by Bonferroni’s test.

SUPPLEMENTARY FIGURE LEGENDS

**Figure S1.** The model of acute cigarette smoke (CS) exposure combined with administration of recombinant fragment of human SP-D (rfhSP-D). Mice were treated with CS for three consecutive days and received 15 μg rfhSP-D one hour before each exposure. Mice were sacrificed 24 h after the last CS exposure.

**Figure S2.** Endogenous SP-D mRNA expression in WT mice is not influenced by either (A) 12 weeks or (B) 3 days of cigarette smoke (CS) exposure. n=5-8.

**Figure S3.** Respiratory mechanics in WT and SP-D-deficient mice after 12 weeks of cigarette smoke (CS) exposure. Subchronic CS exposure did not influence (A) airway resistance (Rrs), (B) airway compliance (Crs), (C) quasi-static compliance (Cst), (D) tissue damping (G), (E) tissue elastance (H) or (F) central airway resistance (Rn), while resulted in significant increase in (G) inspiratory capacity (IC) and (H) estimate of total lung capacity (A) independent of SP-D genotype. I) Area of the pressure-volume curve was significantly increased by CS exposure only in SP-D-deficient mice. n=6-9. *p<0.05, **p<0.01, analyzed by one-way ANOVA followed by Bonferroni’s test.

Figure S4. The levels of long-chain and very long-chain dihydroceramides are increased in SP-D-deficient mice after (A-C) 12 weeks or (D) 3 days of cigarette smoke (CS) exposure. Dihydroceramides (16:0) and (24:1) were undetected in mice subjected to acute CS exposure. n=4-7. **p<0.01, ***p<0.001, ****p<0.0001, analyzed by one-way ANOVA followed by Bonferroni’s test. ND, not detected.
