# Supplementary figures and images for: Surfactant Protein D Deficiency Aggravates Cigarette Smoke-Induced Lung Inflammation by Upregulation of Ceramide Synthesis
Source: Front Immunol. 2018 Dec 18;9:3013. doi: 10.3389/fimmu.2018.03013 (PMC6305334; doi:10.3389/fimmu.2018.03013)

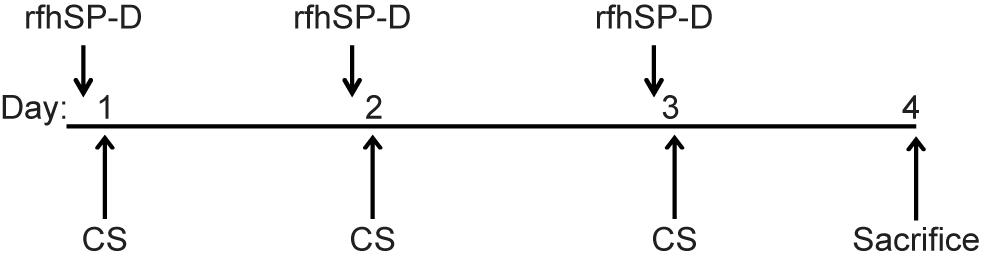

Supplement: Supplementary file 2 [file Image_1.TIF]

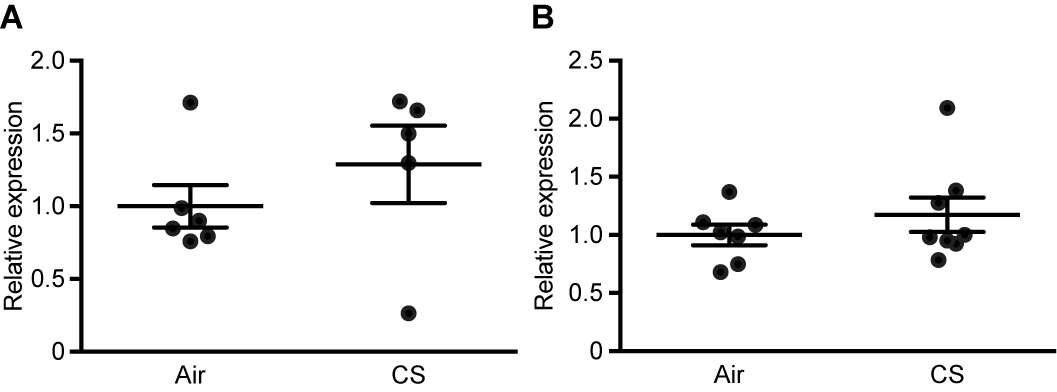

Supplement: Supplementary file 3 [file Image_2.TIF]

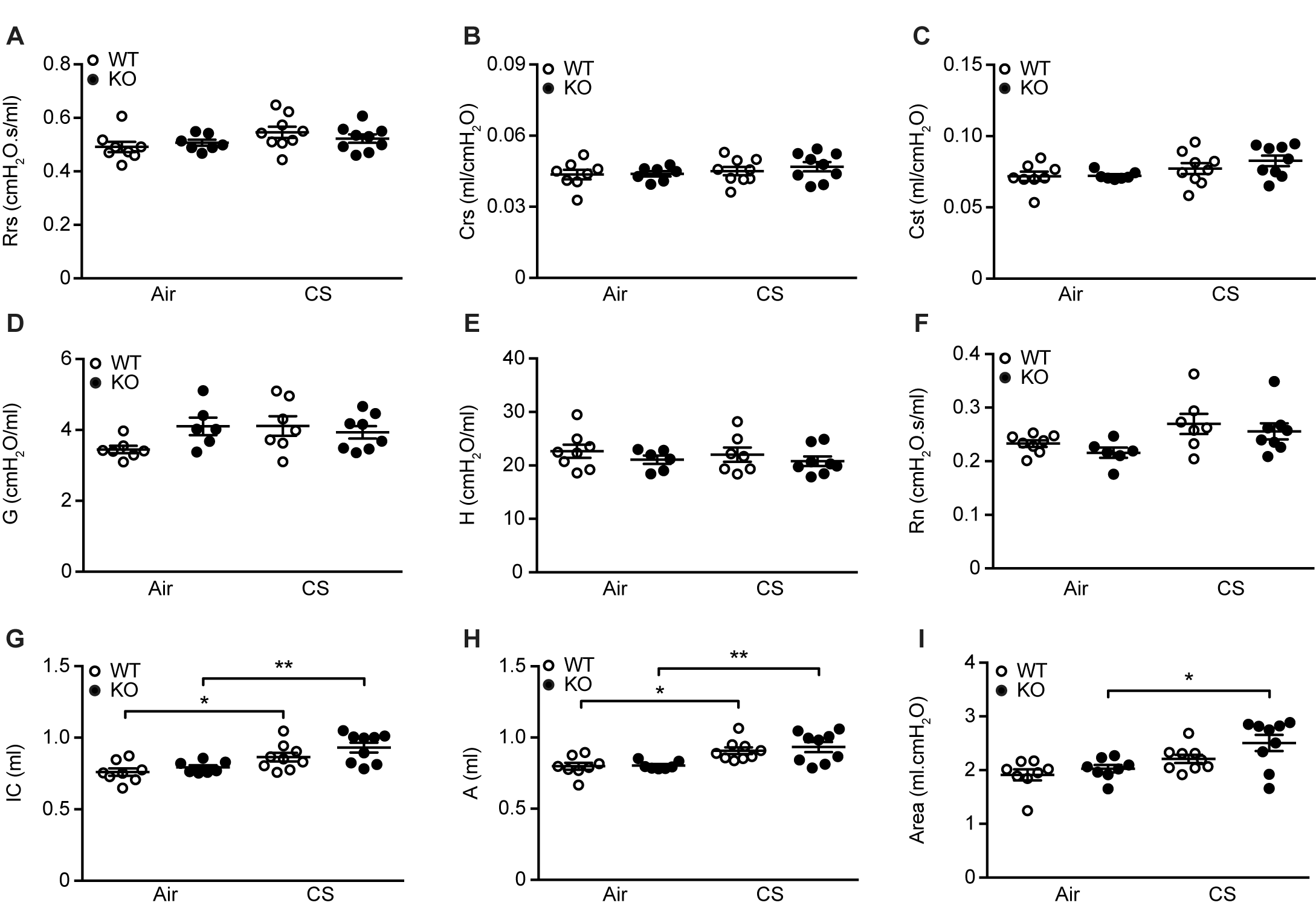

Supplement: Supplementary file 4 [file Image_3.TIF]

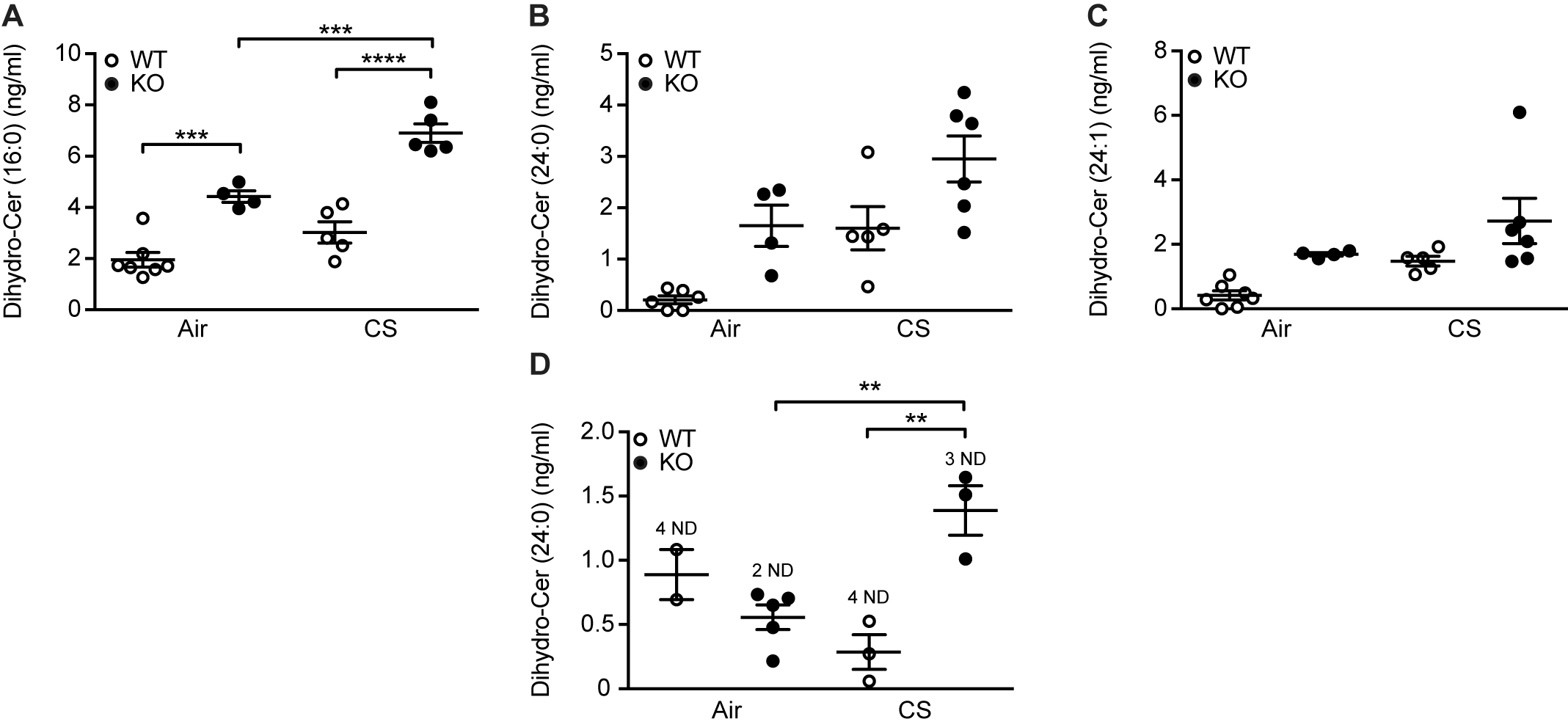

Supplement: Supplementary file 5 [file Image_4.TIF]
